# Directed Evolution of P450 BM3 towards Functionalization of Aromatic O-Heterocycles

**DOI:** 10.3390/ijms20133353

**Published:** 2019-07-08

**Authors:** Gustavo de Almeida Santos, Gaurao V. Dhoke, Mehdi D. Davari, Anna Joëlle Ruff, Ulrich Schwaneberg

**Affiliations:** 1 Lehrstuhl für Biotechnologie, RWTH Aachen University, Worringerweg 3, 52074 Aachen, Germany; 2 DWI-Leibniz-Institut für Interaktive Materialien e.V., Forckenbeckstraße 50, 52074 Aachen, Germany

**Keywords:** protein engineering, directed evolution, P450, monooxygenases aromatic heterocycles, hydroxylation, molecular modeling

## Abstract

The O-heterocycles, benzo-1,4-dioxane, phthalan, isochroman, 2,3-dihydrobenzofuran, benzofuran, and dibenzofuran are important building blocks with considerable medical application for the production of pharmaceuticals. Cytochrome P450 monooxygenase (P450) *Bacillus megaterium* 3 (BM3) wild type (WT) from *Bacillus megaterium* has low to no conversion of the six O-heterocycles. Screening of in-house libraries for active variants yielded P450 BM3 CM1 (R255P/P329H), which was subjected to directed evolution and site saturation mutagenesis of four positions. The latter led to the identification of position R255, which when introduced in the P450 BM3 WT, outperformed all other variants. The initial oxidation rate of nicotinamide adenine dinucleotide phosphate (NADPH) consumption increased ≈140-fold (WT: 8.3 ± 1.3 min^−1^; R255L: 1168 ± 163 min^−1^), total turnover number (TTN) increased ≈21-fold (WT: 40 ± 3; R255L: 860 ± 15), and coupling efficiency, ≈2.9-fold (WT: 8.8 ± 0.1%; R255L: 25.7 ± 1.0%). Computational analysis showed that substitution R255L (distant from the heme-cofactor) does not have the salt bridge formed with D217 in WT, which introduces flexibility into the I-helix and leads to a heme rearrangement allowing for efficient hydroxylation.

## 1. Introduction

Aromatic oxygen-containing heterocycles (O-heterocycles) are significantly abundant in nature as they are present in vitamins, hormones, antibiotics, sugars, pigments, and antioxidants (e.g., vitamin E, coumarin, flavonoids, and isoflavonoids) and are involved in a variety of fundamental biological functions [1,2,3]. These heterocycles and their derivatives are, in most cases, synthesized and functionalized by the traditional chemical route to serve as building blocks for synthetic drugs, pesticides, dyes, and plastics. Benzo-1,4-dioxane, a bicyclic heterocyclic compound consisting of a benzene ring fused to a heterocyclic dioxane ring, represents a series of synthetic and natural compounds [4,5,6,7,8,9,10,11] of considerable medicinal importance with various biological activities [12,13,14] such as antigrastic [15], spasmolytic [16], antipsychotic [17], anxiolytic [18], hepatoprotective [19], or α-adrenergic blocking agent activity [12,20,21]. Functionalization of such heterocycles via chemical oxygenation is still challenging as it involves weary and costly steps that are catalyzed in the presence of expensive and toxic heavy metals [22,23] and often occur with little chemo-, regio-, and/or enantioselectivity leading to sustainability problems [23]. To overcome these challenges, the use of cytochrome P450 monooxygenases (P450s), well known for their ability to hydroxylate non-activated carbon atoms [24,25,26], can provide a powerful tool for the functionalization of aromatic O-heterocycles with high chemo-, regio-, and/or enantioselectivity. In fact, several studies report that P450s can be used to manufacture versatile building blocks for high-value compounds such as pharmaceuticals [25,26,27,28,29]. To that matter, cytochrome P450 from *Bacillus megaterium*, also known as CYP102A1 or P450 *Bacillus megaterium* 3 (BM3), because of its attractive properties such as its self-sufficiency due to a heme and FMN/FAD-containing reductase domains on a single polypeptide, water solubility and relatively high catalytic activity for P450s has been studied extensively and was the subject of intense enzyme engineering campaigns to fully apply and exploit its catalytic power. In fact, throughout the last decades, researchers reported variants with increased activity, better coupling efficiency, expanded substrate scope, and even the ability to perform abiotic reactions [30,31,32,33,34,35,36,37,38,39,40,41]. The application of chemoenzymatic syntheses of aromatic O-heterocycle derivatives in a synthetic late-stage fashion significantly extends the synthetic toolbox, offering chemists an attractive alternative to the conventional chemical strategies [23]. For instance, using P450 oxidation technology, a selective and environmentally friendly route towards the synthesis of 4-hydroxy-α-isophorone on kilogram scale was possible [42]. However, such protein engineering campaigns usually generate thousands of variants, where a major challenge is the development of product-based screening systems to reliably identify better performing catalysts, i.e., the screening system has to be of high throughput, reproducible, and optimized for sensitivity of the desired function. Traditionally, enzyme activity is determined in 96-microtiter plates (MTPs) using either crude cell lysates or purified enzyme to perform product-based colorimetric or fluorometric assays (e.g., 4-aminoantipyrine for phenolic compound detection [43], NpCN for the detection of specific hydroquinones [44], pNTP for styrene epoxidation [45], or fluorescence for the detection of steroid hydroxylation [46]). A generally applicable and emerging possibility is 96 multiplex-capillary electrophoresis (CE), which has been added to the range of suitable screening systems for P450-directed evolution campaigns [47]. It is a powerful, versatile, and automated technique for the separation and analysis of charged substances and biological macromolecules such as amino acids, peptides and proteins, chiral drugs, whole cells, and virus particles to name a few [48,49]. Furthermore, depending on the analyte and application, different detection systems can be coupled (UV-vis spectrophotometric detection, laser-induced fluorescence (LIF), contactless conductivity detection (CCD), or even mass spectrometers (MS)) [48].

**Figure 1:**
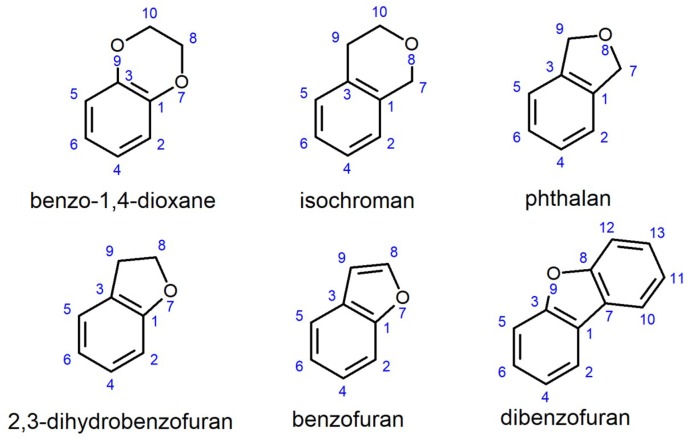
2D chemical structure of the tested aromatic O-heterocycles.

The aim of this study was to explore the potential of P450 BM3 in synthetizing hydroxylated aromatic O-heterocycles that can be used as building blocks for the production of high-value compounds. Screening of mutant libraries in a KnowVolution-like approach [45] was used to identify the key position 255, which significantly improved the hydroxylation activity towards the substrate benzo-1,4-dioxane. The substrate scope of the obtained P450 BM3 R255L and R255G variants was investigated by determining the catalytic performance towards phtalan, isochroman, 2,3-dihydrobenzofuran, benzofuran, and dibenzofuran (Figure 1).

## 2. Results and Discussion

Functionalization of benzo-1,4-dioxane, phtalan, isochroman, benzofuran, 2,3-dihydrobenzofuran, and dibenzofuran via enzymatic hydroxylation can provide novel synthetic routes to produce pharmaceutical precursors in a selective and environmentally friendly way. In the first part of this section, we describe the use of a 4-aminoantipyrine (4-AAP) assay in combination with CE for a product-based screening of 2,3-dihydro-1,4-benzodioxin-5-ol and 2,3-dihydro-1,4-benzodioxin-6-ol. The second part reports the protein engineering approach used to improve the hydroxylation of benzo-1,4-dioxane by P450 BM3. The third part focuses on kinetic characterizations and the improved activity in hydroxylating O-heterocycles. Finally, the identified beneficial amino acid substitutions in the improved P450 BM3 variants were analyzed by molecular dynamics simulations to gain molecular understanding.

### 2.1. Development of 4-AAP and CE Screening Systems for Product-Based Quantification of 2,3-Dihydro-1,4-Benzodioxin-5-ol and 2,3 Dihydro-1,4-Benzodioxin-6-ol

**Figure 2:**
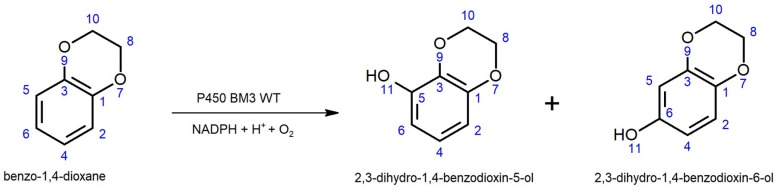
The hydroxylation of benzo-1,4-dioxane by cytochrome P450 monooxygenase (P450) *Bacillus megaterium* 3 (BM3) wild type (WT) leads to the formation of 2,3-dihydrobenzo-1,4-dioxin-5-ol and 2,3-dihydrobenzo-1,4-dioxin-6-ol at a 70/30 ratio.

The two major products of the biotransformation of benzo-1,4-dioxane with P450 BM3 wild type (WT) were identified to be 2,3-dihydro-1,4-benzodioxin-5-ol and 2,3-dihydro-1,4-benzodioxin-6-ol, in a 70/30 ratio (Figure 2). Since hydroxylation occurred on the benzene ring, an assay showing color formation in the presence of phenolic compounds would offer itself as a simple means for high-throughput screening. 4-aminoantipyrine (4-AAP) is a compound that was first introduced for the reliable and sensitive detection of phenols (μg/L) in aqueous solution assays in the 1940s [50].

**Figure A1:**
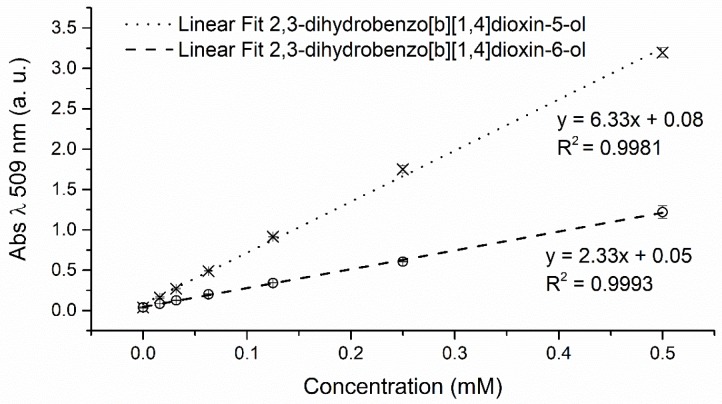
The 4-AAP linear detection range of 2,3-dihydrobenzo-1,4-dioxin-5-ol and 2,3-dihydrobenzo-1,4-dioxin-5-ol in a 96-well MTP format.

**Figure A2:**
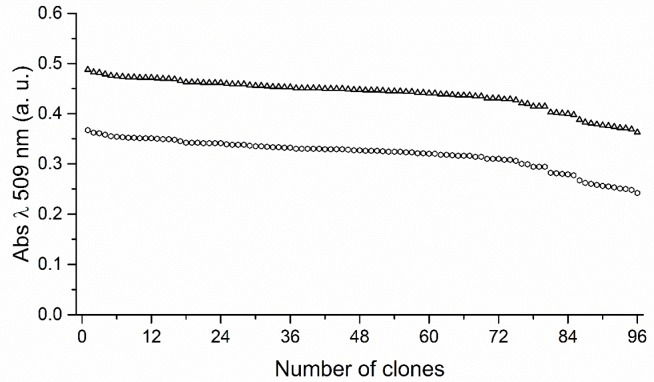
Standard deviation of the 4-AAP assay with P450 BM3 WT. Measured absorption values at λ 509 nm in descending order of P450 BM3 WT catalyzed conversion of benzo-1,4-dioxane in a 96-well plate. The apparent standard deviation (6.8%) is depicted with white triangles. The white hexagons show the true standard deviation (9.4%) after subtraction of the empty vector background.

**Figure A3:**
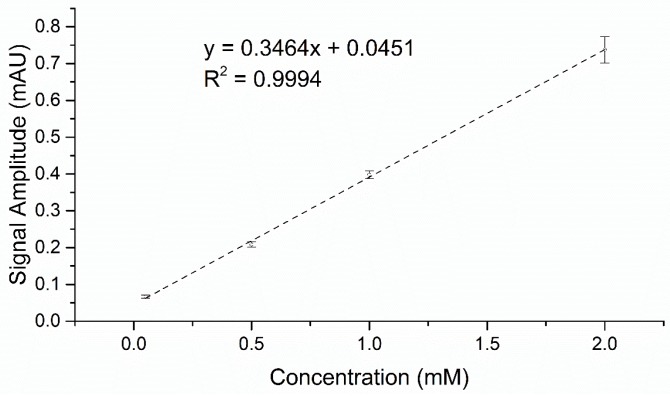
The CE linear detection range of 2,3-dihydrobenzo-1,4-dioxin-5-ol in a 96-well MTP format.

**Figure A4:**
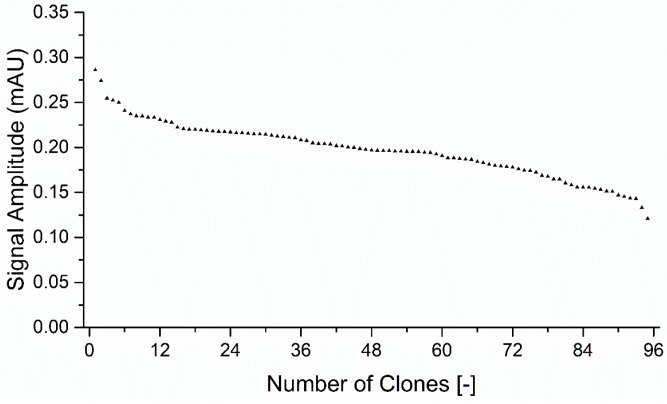
Standard deviation of the CE with P450 BM3 WT. Detected signal amplitude (mAU) at λ 214 nm in descending order of P450 BM3 variant WT catalyzed conversion of benzo-1,4-dioxane in a 96-well plate. The standard deviation (15.6%) is depicted with white triangles.

The interaction between phenols and 4-AAP through oxidative coupling leads to an extended conjugated electron system with strong absorbance at λ 509 nm [43,50]. The 4-AAP assay conditions were adjusted from Wong et al. (2005) for application in phosphate buffer (KPi 50 mM, pH 7.5) and in MTP format. Under the new conditions, 2,3-dihydro-1,4-benzodioxin-5-ol and 2,3-dihydro-1,4-benzodioxin-6-ol concentrations showed a linear response from 16 to 500 μM at λ 509 nm (Figure A1). We applied the nicotinamide adenine dinucleotide phosphate (NADPH) depletion assay [51] in combination with the 4-AAP assay to assess both NADPH depletion rates and total product formation. The standard deviation of the 4-AAP assay after full depletion of NADPH was 6.8% using the WT. After subtraction of the background (EV lysate), a true standard deviation of 9.6% was obtained (Figure A2). Standard deviations below 15% are routinely employed in successful directed evolution campaigns [43,52]. The 4-AAP assay can detect phenolic compounds, but it cannot detect products hydroxylated at the heterocycle ring. To overcome this limitation, we used 96-well CE for the separation and UV detection of products hydroxylated at the heterocycle ring since the benzene ring in benzo-1,4-dioxane has conjugated π-electron systems that strongly absorb in the UV range. The conditions used for CE separation and detection via UV spectroscopy were adapted from Anna et al. (2019) and used in parallel with the NADPH depletion assay. However, the NADPH concentration used (200 μM) was not sufficient to detect a significant formation of 2,3-dihydro-1,4-benzodioxin-5-ol and 2,3-dihydro-1,4-benzodioxin-6-ol. To achieve higher product amounts, an NADPH regeneration solution containing glucose dehydrogenase (GDH) (3 U/mL), glucose (60 mM) and catalase (1200 U/mL) was used. We investigated different NADPH regeneration times (0.5–20 h), and 4 h turned out to be suitable for complementing the rescreening. Under these new conditions, the CE detector showed a linear response between 50 µM and 2 mM (Figure A3) with a standard deviation of 15.6% after 4 h of reaction using the WT (Figure A4).

### 2.2. P450 BM3 Library Generation and Screening

**Figure 3:**
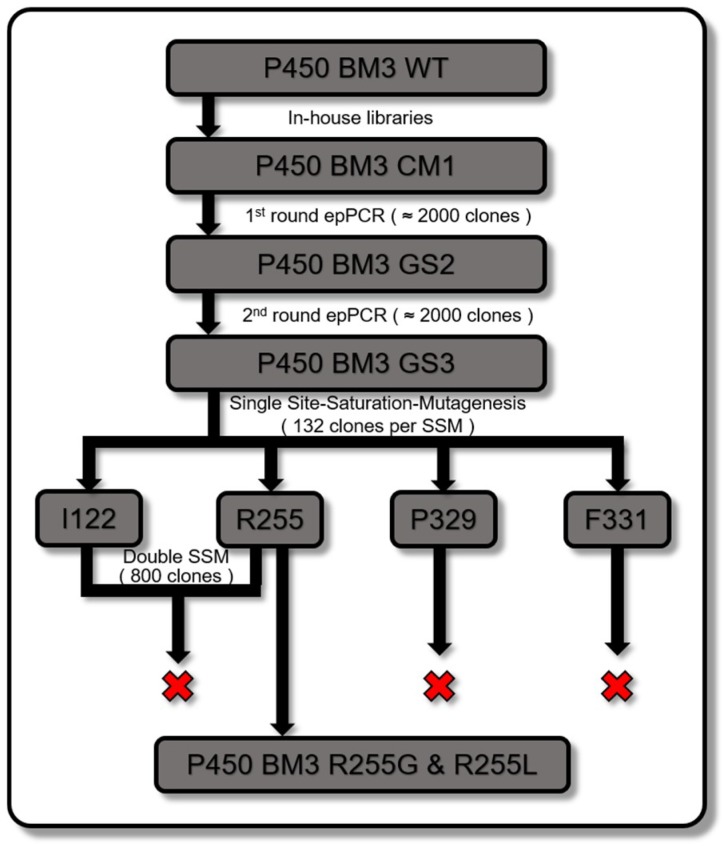
Summary of P450 BM3 engineering strategy. Starting from the top to the bottom, in-house libraries of P450 BM3 were screened yielding P450 BM3 CM1 (R255P/P329H), which was subjected to epPCR, and P450 BM3 GS2 (R255S/P329H/F331L) was generated. P450 BM3 GS2 was subjected to another epPCR round yielding P450 BM3 GS3 (I122V/R255S/P329H/F331L). P450 BM3 WT was subjected to single site saturation mutagenesis (SSM) in the four identified positions and double SSM at positions I122 and R255, which led to the most active variants, P450 BM3 R255G and R255L.

**Figure A5:**
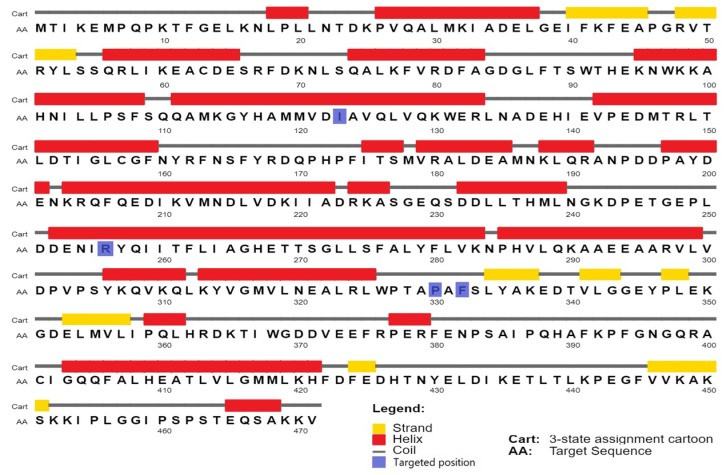
P450 BM3 WT sequence annotated with targeted SSM positions and secondary structure. Yellow boxes as β-strand, red boxes as α-helix, gray boxes as coil, and blue boxes as the targeted position for SSM. Image prepared with PSIRED Server [80].

**Figure A6:**
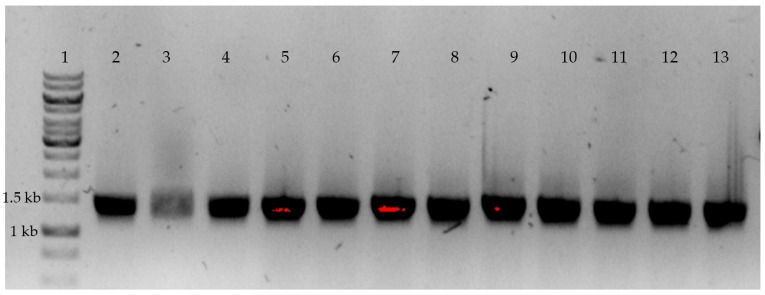
Agarose gel (1% (*w*/*v*)) confirmation of epPCR products. Amplifications using in-house *Taq* DNA polymerase, 1- MWM 1 kb GeneRuler, 2 to 12–0.05 mM heme domain (1452 bp).

The P450 BM3 engineering strategy is summarized in Figure 3, where screening of previously prepared in-house epPCR and site-saturation-mutagenesis (SSM) libraries of P450 BM3 WT [44,47,53] yielded P450 BM3 CM1 (R255P/P329H), which was subjected to two rounds of epPCR to identify additional beneficial positions. This led to the identification of four positions in total (I122, R255, P329, and F331) that were selected for individual site saturation mutagenesis (SSM) using WT as template (Figure A5). Briefly, epPCR using a MnCl_2_ concentration of 0.05 mM was performed on the heme domain of P450 BM3 CM1 and confirmed by agarose gel electrophoresis (Figure A6). The P450 BM3 CM1 (R255P/P329H) gene libraries were cloned into the vector backbone (pALXtreme-1a) via PLICing and subsequently transformed into chemically competent *Escherichia coli* BL21-Gold (DE3) lacI^Q1^ cells.

**Figure 4:**
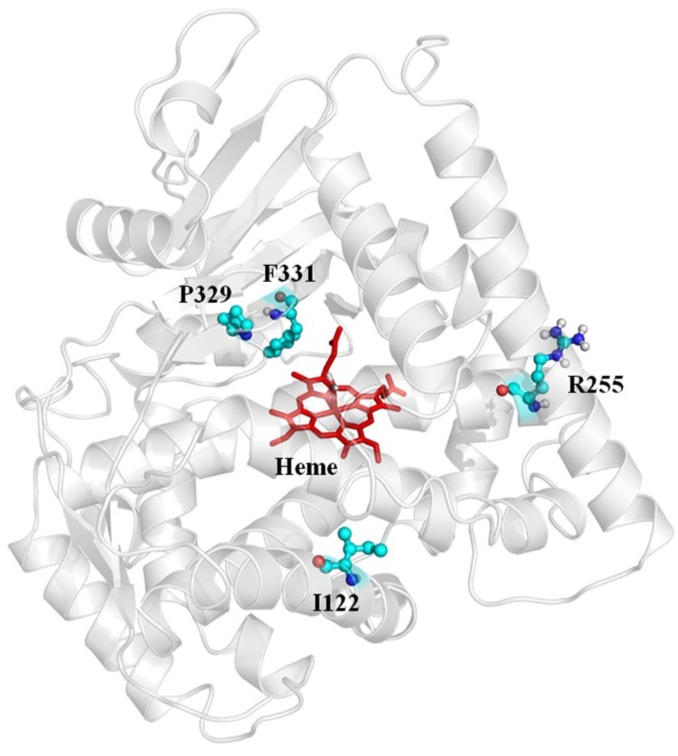
The crystal structure of P450 BM3 WT illustrating the 4 identified positions (I222, R255, P329, and F331) from the two rounds of epPCR. The positions selected are represented as ball and sticks. The heme cofactor is depicted in red sticks.

**Figure A7:**
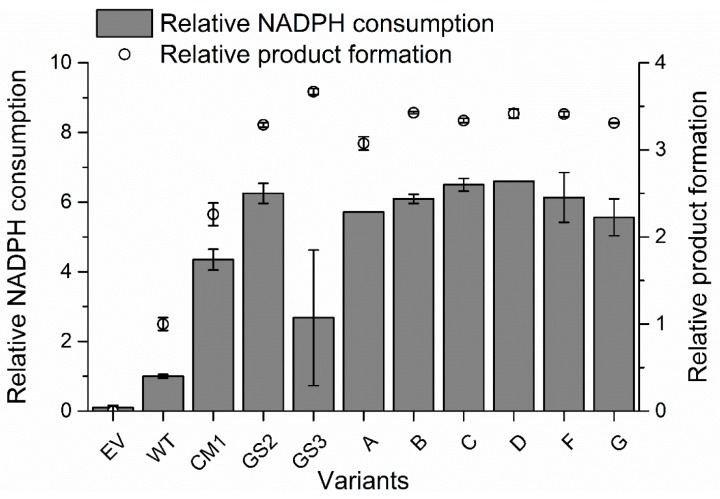
Comparison between P450 BM3 WT and resulting variants from the 2 rounds of epPCR of NADPH consumption rate (bars) and product formation (circles) using a 4-AAP assay. Error bars represent one SD of the mean from three replicates. CM1 (R255P/P329H), GS2 (R255S/P329H/F331L), and GS3 (I122V/R255S/P329H/F331L).

The percentage of active clones/mutational load was adjusted to the 96-well microtiter plate (MTP) screening format, to efficiently screen mutant libraries and minimize screening efforts. We aimed for 40% to 60% of active clones per MTP. The percentage of active clones was determined by expressing 88 clones in 96-well MTPs and subsequent activity determination (NADPH depletion assay and 4-AAP assay). The variants were considered active if they exhibited a higher NADPH consumption rate than that of the empty vector. Screening of each P450 BM3-epPCR library (0.05 mM MnCl_2_) was performed via NADPH depletion assay in combination with a 4-AAP assay in a 96-well format. The lysate of *E. coli* BL21-Gold (DE3) lacI^Q1^ expressing P450 BM3 WT (WT) in pALXtreme-1a served as a positive control and the lysate of *E. coli* BL21-Gold (DE3) lacI^Q1^-pALXtreme-1a (EV) as a negative control in each MTP (in quadruplicates). Activity was determined by measuring the decrease in absorbance (NADPH depletion) and taken as an absolute value of the slope, plus measuring the absorbance at λ 509 nm after performing the 4-AAP assay. Variants exhibiting significantly higher absolute values of the slope (i.e., activity) and/or higher absorbance at λ 509 nm (after 4-AAP assay) than the WT were selected for rescreening. In total, nearly 4000 clones from the two rounds of epPCR were screened, and the most promising variants were selected for re-screening. Re-screening results revealed that from each round of epPCR, the variants exhibited improved activity and/or higher product formation in comparison to the WT (Figure A7). The selected variant P450 BM3 GS3 (I122V/R255S/P329H/F331L) exhibited the highest product formation and was subsequently sent for sequencing analysis. To guide the recombination of beneficial substitutions, the crystal structure of P450 BM3 WT (PDB ID: 1BU7 [54]) was visually inspected to locate substitutions (Figure 4).

**Figure 5:**
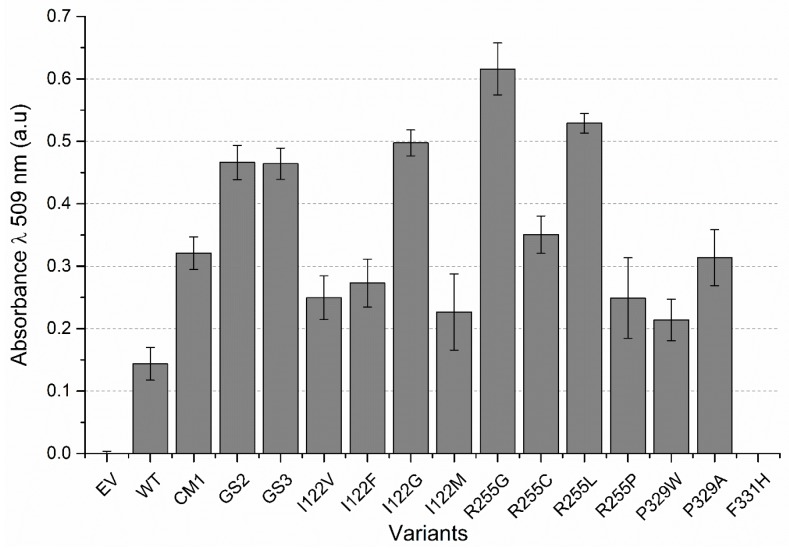
Comparison of 2,3-dihydrobenzo-1,4-dioxin-5-ol and 2,3-dihydrobenzo-1,4-dioxin-6-ol formation by the colorimetric 4-aminoantipyrine (4-AAP) assay (λ 509 nm). Results show the best-performing P450 BM3 from SSM at the positions 122, 255, 329, and 331. Error bars represent one SD of the mean from seven replicates. CM1 (R255P/P329H), GS2 (R255S/P329H/F331L), and GS3 (I122V/R255S/P329H/F331L). EV—negative lysate control.

All four positions (I122, R255, P329, and F331) were selected for individual site saturation mutagenesis (SSM) using the WT as a template. Each SSM library was screened using the 4-AAP screening assay in the same way as performed for the epPCR libraries. After the screening of 528 clones, 11 P450 BM3 WT-SSM variants showed significantly increased activity in comparison to WT (Figure 5) and were selected for rescreening by CE to evaluate the formation of additional products. The rescreening revealed that the most active variants had substitutions at position 122 and 255. Therefore, a double SSM library was prepared (P450 BM3 WT-dSSM), but no variant with a relative product formation higher than P450 BM3 R255G (R255G) was found after screening of nearly 800 clones. Variants R255G and R255L exhibited at least a 4.0- and 3.5-fold improvement, respectively, in total product formation (analyzed via 4-AAP assay). Furthermore, no additional product formation was detected (analyzed via CE, 8). These results led to the selection of R255G and R255L variants for further characterization.

### 2.3. Characterization of P450 BM3 WT and Variants R255G and R255L in Respect to Hydroxylation of the Six Selected O-Heterocycles

**Figure 6:**
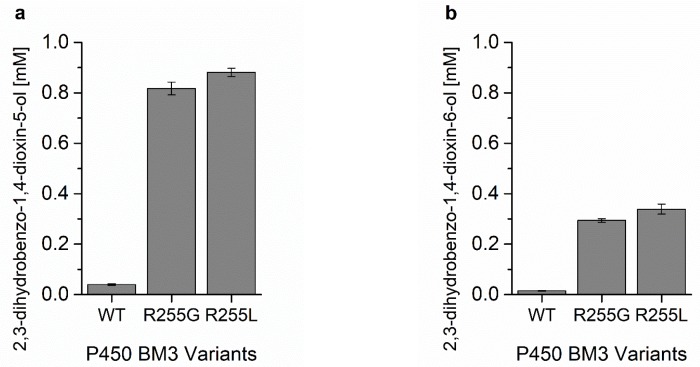
Product formation of different P450 BM3 variants measured with GC-FID. (**a**) 2,3-dihydrobenzo-1,4-dioxin-5-ol detected by GC-FID after 1 h of benzo-1,4-dioxane conversion. (**b**) 2,3-dihydrobenzo-1,4-dioxin-6-ol detected by GC-FID after 1 h of benzo-1,4-dioxane conversion.

**Figure A9:**
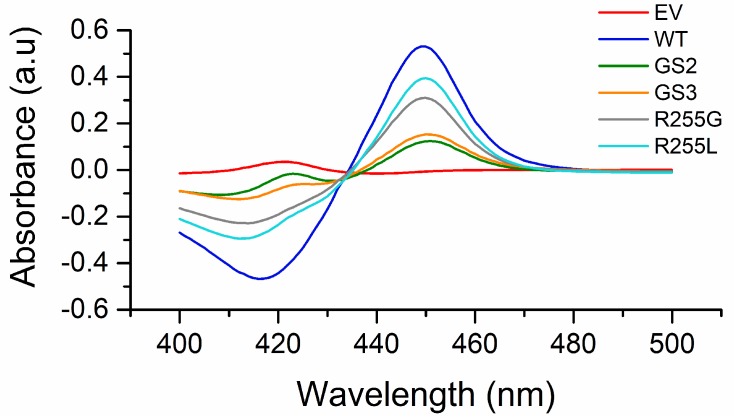
Absorption spectra for the produced variants of P450 BM3 in their ferrous carbon monoxide complex. A typical absorption spectrum for the WT of P450 BM3 is shown in blue. EV—empty vector (negative control), WT—wild-type, GS2 (R255S/P329H/F331L), and GS3 (I122V/R255S/P329H/F331L).

**Figure A10:**
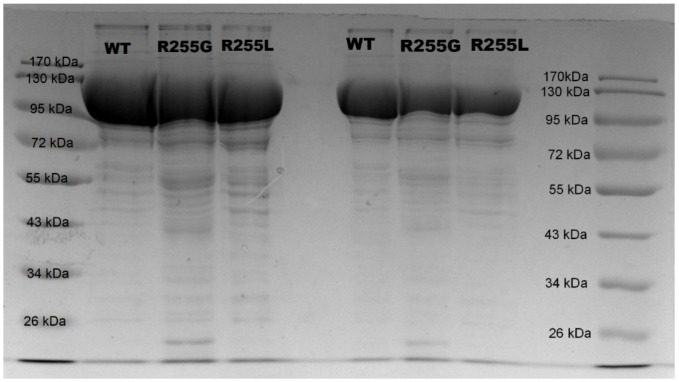
SDS-PAGE (10% Acrylamide) analysis of the purified P450 BM3 WT and variants R255G and R255L. The molecular weight of P450 BM3 WT and variants proteins are ca. 118 kDa.

The obtained P450 BM3 R255G (R255G) and P450 BM3 R255L (R255L) variants were expressed, purified, quantified (Figure A9 and Figure A10), and characterized in detail by performing conversions of benzo-1,4-dioxane in 1 mL volume in the presence of a cofactor regeneration system (GDH). Product formation was assessed with GC-FID. We observed solubility issues for benzo-1,4-dioxane as its limit was 1.2 mM when using ethanol as a co-solvent. After 1 h of conversion, under constant NADPH regeneration, R255G and R255L produced 0.80 ± 0.02 mM and 0.86 ± 0.02 mM of 2,3-dihydrobenzo-1,4-dioxin-5-ol, respectively, whereas P450 BM3 WT (WT) produced 0.040 ± 0.003 mM (Figure 6a). This is a ≈20- and ≈22-fold improvement over WT for R255G and R255L.

**Figure 7:**
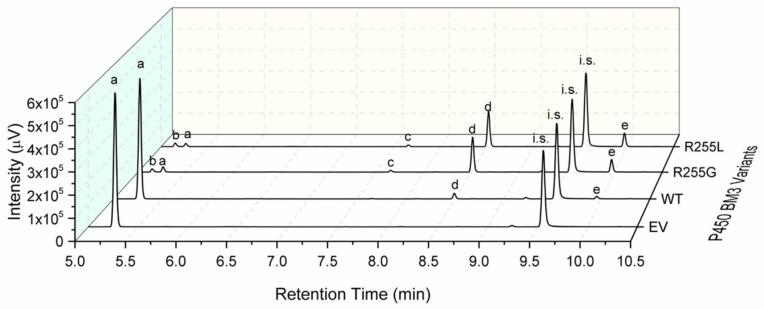
Product analysis using GC-FID after conversion of benzo-1,4-dioxane with lysate from EV (negative control), P450 BM3 WT, and from variants R255G and R255L. The data was obtained from 1 h conversion reactions employing glucose dehydrogenase (GDH) for efficient NADPH cofactor regeneration. a—benzo-1,4-dioxane, d—2,3-dihydrobenzo-1,4-dioxin-5-ol, e—2,3-dihydrobenzo-1,4-dioxin-6-ol, i.s.—cyclododecanol, b, c—unknown product. An improved 2,3-dihydrobenzo-1,4-dioxin-5-ol formation is visible with R255G and R255L compared to WT.

The formation of the 2,3-dihydrobenzo-1,4-dioxin-6-ol was also improved by R255G and R255L (Figure 6b), and the ratio of their formation remained the same as for the WT (73 ± 1/27 ± 1). The coupling efficiencies of R225G and R255L were similar, 23.7 ± 0.5% and 25.7 ± 1.0% respectively (Table 1) and significantly improved when compared to the WT (8.8 ± 0.1%). Furthermore, the variants R255G and R255L reached a total product concentration of 121 mg/L and 131 mg/L, corresponding to a total turnover number (TTN) of 798 ± 24 and 860 ± 15, respectively. Indeed, GC-FID analysis revealed that R255L is able to convert over 95% of the loaded substrate (1.2 mM) in 1 h of reaction, whereas the WT converts < 7%, making R255L a better catalyst for benzo-1,4-dioxane hydroxylation, as shown in Figure 7.

**Figure A11:**
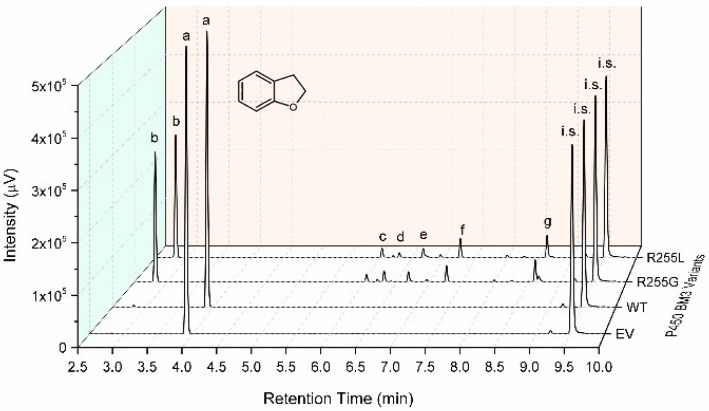
Product analysis via GC-FID after conversion of 2,3-dihydrobenzofuran with EV, P450 BM3 WT, and variants R255G and R255L. The data was obtained from 1 h conversion reactions employing GDH for efficient NADPH cofactor regeneration. a—2,3-dihydrobenzofuran, i.s.—cyclododecanol, b, c, d, e, f, g—unknown product. A total 2,3-dihydrobenzofuran depletion is visible with R255G and R255L, whereas the WT does not convert the substrate.

**Figure A12:**
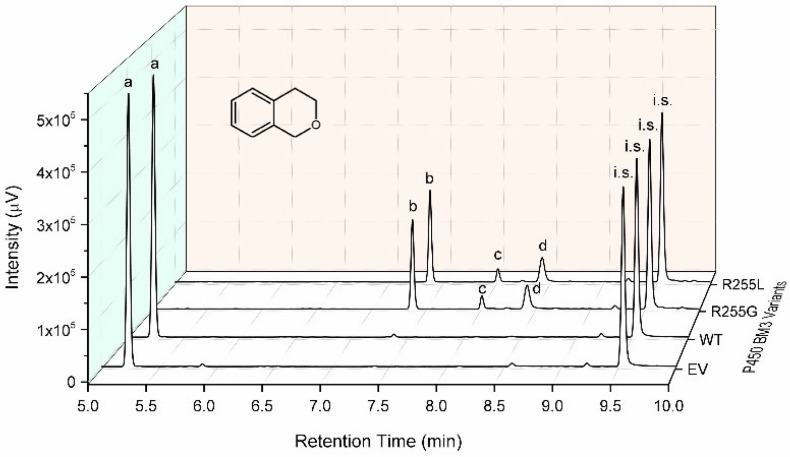
Product analysis via GC-FID after conversion of isochroman with lysate from EV (negative control), P450 BM3 WT, and from variants R255G and R255L. The data was obtained from 1 h conversion reactions employing GDH for efficient NADPH cofactor regeneration. a—isochroman, i.s.—cyclododecanol, b, c, d—unknown product. A complete isochroman depletion is visible for R255G and R255L, whereas WT is virtually unable to convert isochroman (≤1.5 ± 0.2% conversion).

**Figure A13:**
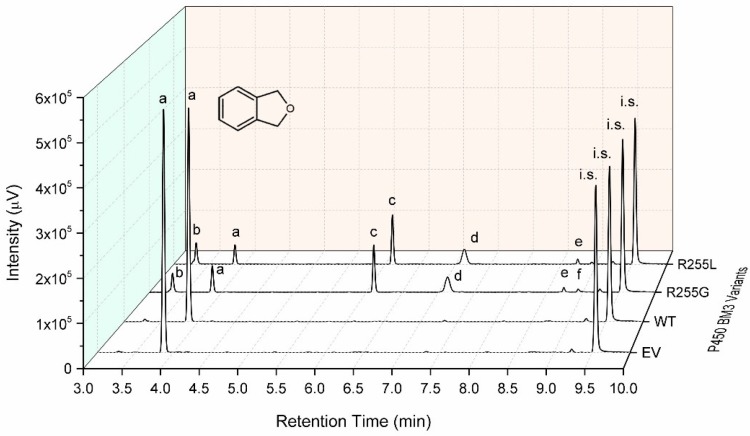
Product analysis via GC-FID after conversion of phthalan with lysate from EV (negative control), P450 BM3 WT, and from variants R255G and R255L. The data was obtained from 1 h conversion reactions employing GDH for efficient NADPH cofactor regeneration. a—phthalan, i.s.—cyclododecanol, b, c, d, e, f—unknown product. A nearly 90% phthalan depletion is visible for both R255G and R255L, whereas WT can only convert 6 ± 1%.

**Figure A14:**
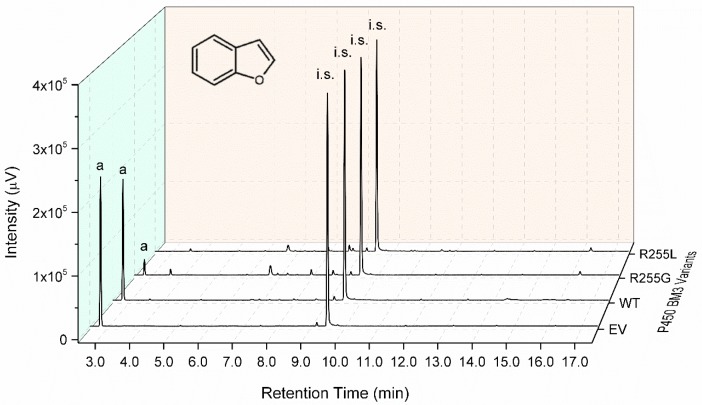
Product analysis via GC-FID after conversion of benzofuran with lysate from EV (negative control), P450 BM3 WT, and from variants R255G and R255L. The data was obtained from 1 h conversion reactions employing GDH for efficient NADPH cofactor regeneration. a—benzofuran, i.s.—cyclododecanol. A nearly 90 ± 2% benzofuran depletion is visible with R255G and complete depletion with R255L, whereas WT is only able to convert 19 ± 6%.

**Figure A15:**
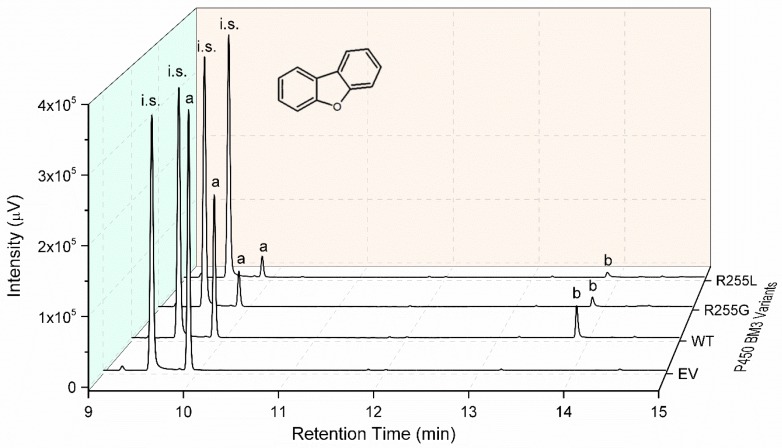
Product analysis via GC-FID after conversion of dibenzofuran with lysate from EV (negative control), P450 BM3 WT, and from variants R255G and R255L. The data was obtained from 1 h conversion reactions employing GDH for efficient NADPH cofactor regeneration. a—dibenzofuran, b—unknown product, i.s.—cyclododecanol. A conversion of 77 ± 0% and 85 ± 3% of dibenzofuran is visible for R255G and R255L, respectively, whereas WT is only able to convert 16 ± 4%.

From the latter, we can also observe the detection of two additional products that are likely to be the hydroxylated product at the dioxane ring (2,3-dihydro-1,4-benzodioxin-2-ol) and its dehydrated form 1,4-benzodioxin due to the heating program in the GC-FID. However, the amounts produced were not sufficient for identification using GC-MS (data not shown). We investigated the influence that the residue R255 might have on the ability of P450 BM3 to convert other O-heterocycles, namely phthalan, isochroman, benzofuran, 2,3-dihydrobenzofuran, and dibenzofuran. Indeed, during a 1 h reaction, the variants R255G and R255L achieved high conversion of phtalan (R255G: 82 ± 7%; R255L: 90 ± 1%) and dibenzofuran (R255G: 77 ± 0%; R255L: 85 ± 3%) and full conversion of isochroman (R255G and R255L: ≥ 99%), 2,3-dihydrobenzofuran (R255G and R255L: ≥ 99%), and benzofuran (R255G: 90 ± 2%; R255L: ≥ 99%) (analyzed via GC-FID: Figure A11, Figure A12, Figure A13, Figure A14 and Figure A15). In contrast, P450 BM3 WT had low conversion numbers for phtalan (≤7%), isochroman (≤2%), 2,3-dihydrobenzofuran (≤1%), benzofuran (19 ± 6%), and dibenzofuran (16 ± 4%).

### 2.4. Rationale behind the Activity Improvement of R255G and R255L Variants over the WT

**Figure 8:**
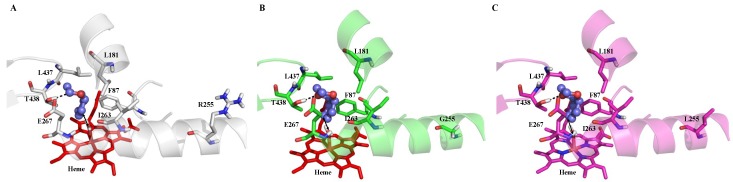
Molecular docking pose of benzo-1,4-dioxane in the active site of WT, R255G, and R255L (**A**) WT (binding energy: −5.63 kcal/mol), (**B**) R255G variant (binding energy: −5.66 kcal/mol), and (**C**) R255L variant (binding energy: −5.66 kcal/mol). Reciprocal arrows indicate the closest distance between the iron-bound water ligand and C5 atom of benzo-1,4-dioxane (WT: 5.14 Å, R255G and R255L: 4.90 Å). Substrate benzo-1,4-dioxane is depicted as a ball and stick, whereas all the active site residues including heme are depicted as sticks. Hydrogen bond between substrate and T438 is represented as a black dotted line.

Computational analysis was carried out using benzo-1,4-dioxane as substrate. Molecular docking studies were performed to investigate the interactions of benzo-1,4-dioxane with P450 BM3’s active site. Figure 8 shows the most probable binding orientation of benzo-1,4-dioxane in the binding pocket of WT, R255G, and R255L. The docking simulations revealed that in all three cases, the substrate binds in a similar manner (close to the water molecule covalently bound to the central iron atom).

**Figure 9:**
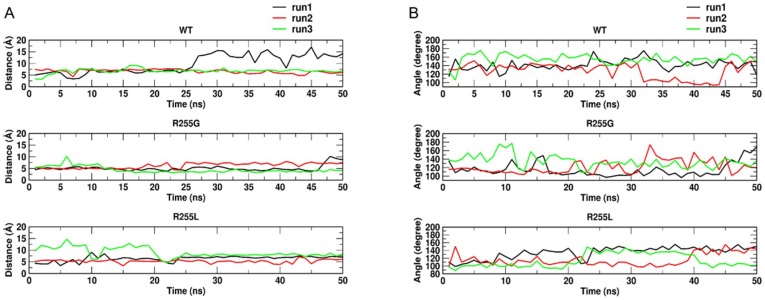
(**A**) Calculated distance between C5-atom of substrate and iron-bound oxygen species. (**B**) Calculated angle between benzo-1,4-dioxane substrate and heme required for the hydroxylation of benzo-1,4-dioxane in WT and variants R255G and R255L along three independent 50 ns molecular dynamics (MD) simulation trajectories.

**Figure A16:**
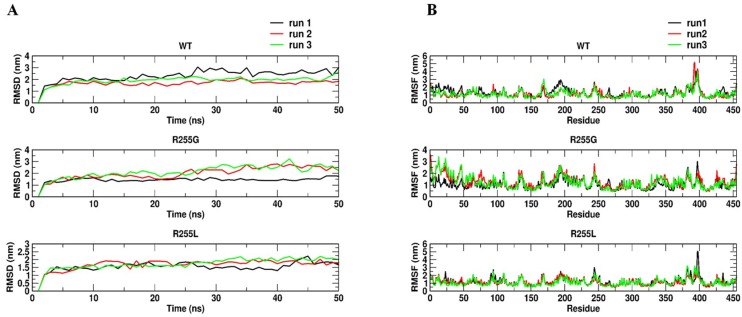
Calculated (**A**) root mean square deviation (RMSD) and (**B**) root mean square fluctuation (RMSF) per residue of P450 BM3 WT and P450 BM3 variants (R225L and R255L) throughout three independent MD simulation runs (I-helix: residues 245 to 283).

Furthermore, there were no significant differences in the binding energies, although a slight change in the distance between the substrate-closest C atom (C5) and the iron-bound water molecule was observed in both R255G and R255L (≈4.90 Å) vs. WT (≈5.14 Å). This observation suggests a slow activation of the substrate by WT despite the binding of benzo-1,4-dioxane; however, the influence of the residue R255 on the activity remained unresolved. This residue is located on the distal side of the I-helix and far away from the substrate-binding pocket. It is known that the I-helix is the most prominent structural component in P450, providing a backbone for heme arrangement and the remaining chain [55]. Therefore, to get a deeper molecular understanding of the influence of R255 residue on P450 BM3 activity, molecular dynamics (MD) simulations were carried out. The enzyme-substrate complex of benzo-1,4-dioxane for WT and variants were further subjected to MD simulations to analyze the stability and orientation as well as the nature and energetics of substrate binding. Root mean square deviation (RMSD) (Figure A16) analysis shows the stability of substrate-enzyme complex throughout the MD simulations. Substitution of R255 by either glycine or leucine indeed introduced flexibility in the I-helix, as evidenced by root mean square fluctuation (RMSF) per residue analysis. From MD simulations, it was observed that in WT, benzo-1,4-dioxane initially stays in close contact with the heme but moves away afterwards (Figure 9A). 

**Figure 10:**
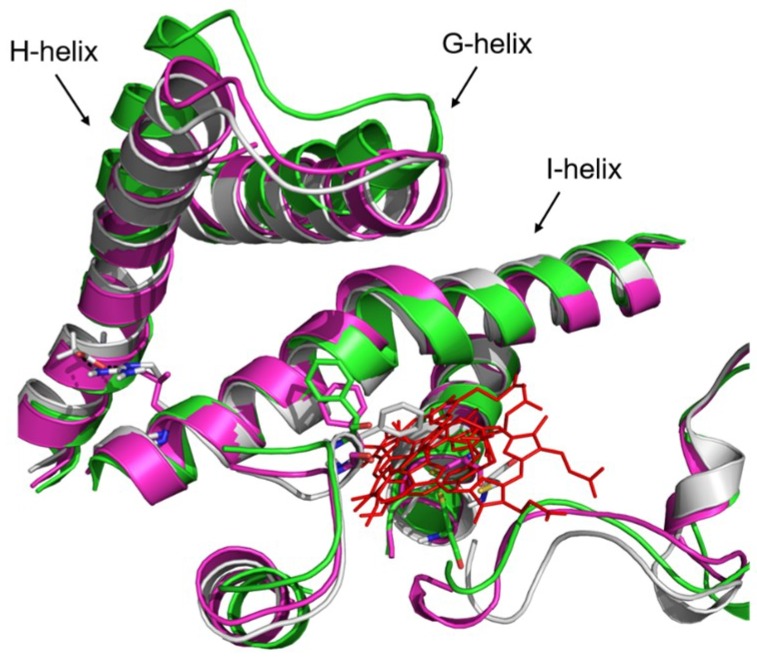
Cartoon representation of the structural alignment of P450 BM3 WT (grey) and P450 BM3 variants (R255G in green and R255L in magenta). Heme is depicted in red lines and residue F87 in sticks. A substantial rearrangement of the G-, H-, and I-helixes is observed. The models of the P450 BM3 variants R255G and R255L were constructed using the swap function in YASARA Structure Version 17.4.17 and optimized using the SCWRL rotamer library search for the designated substitutions.

By contrast, in R255G and R255L variants, the C5-atom on which the actual hydroxylation takes place remains in close contact with the iron-bound water molecule, allowing for hydroxylation to occur. Indeed, a recent study on isophorone hydroxylation using P450-WAL [56] showed that the ideal distance and angle between isophorone and heme for catalytically competent hydroxylation should be approximately 3 Å and 109–149 degrees, respectively, which is well supported by our R255G and R255L variant simulations (Figure 9). Additionally, R255 is important for the structural rigidity of the I-helix due to the formation of a salt bridge with D217 in WT. Hence, substituting R255 with either G or L, this salt-bridge will not be formed, leading to increased flexibility in the I-helix of R255G and R255L variants (Figure A16). A structural rearrangement in the heme-binding domain was also observed, especially in residue F87 (Figure 10), which causes the substrate to adapt and maintain the catalytically competent orientation. Indeed, throughout 50 ns of MD simulations, benzo-1,4-dioxane persistently kept a distance ≈ 3 Å and an angle of 109–149 degrees required for hydroxylation, as shown in Figure 9. This rearrangement could thus lead to the improved performance of P450 BM3 R255G and R255L towards benzo-1,4-dioxane. 

In conclusion, protein engineering by directed evolution and site saturation mutagenesis of identified positions revealed the important role of position R255 in boosting the catalytic performance of P450 BM3 towards aromatic O-heterocyclic compounds. The increased performance was not limited to the evolution substrate (i.e., benzo-1,4-dioxane; R255G: ≈ 90%; R255L: ≈ 95%; WT: ≤ 7%), and indeed, similar improvements in conversions were achieved for phthalan (R255L and R255G: ≈ 90%; WT: ≤ 2%), isochroman (R255L and R255G: ≥ 99%; WT: ≤ 2%), 2,3-dihydrobenzofuran (R255L and R255G: ≥ 99%; WT: ≤ 2%), benzofuran (R255G: 90 ± 2%; R255L: ≥ 99%; WT: 19 ± 6%), and dibenzofuran (R255G: 77 ± 0%; R255L: 85 ± 3%; WT: 16 ± 4%). The P450 BM3 variant R255L, is ca. 22 times more active than the WT in hydroxylating benzo-1,4-dioxane. This substitution has a drastic influence on the catalytic activity of P450 BM3 as compared to the WT, increasing the coupling efficiency (25.7 ± 1.0% vs. 8.8 ± 0.1%), NADPH oxidation rate (1168 ± 163 min^−1^ vs. 8.3 ± 1.3 min^−1^), and TTN (860 ± 15 vs. 40 ± 3). Computational analysis reveals that breaking a salt bridge (formed between R255 and D217 in WT) introduces flexibility in the I-helix and leads to a productive heme rearrangement, thus improving benzo-1,4-dioxane hydroxylation.

The improvement observed in P450 BM3 R255G and R255L towards benzo-1,4-dioxane, phtalan, isochroman, benzofuran, 2,3-dihydrobenzofuran, and dibenzofuran provides useful enzymatic routes to produce pharmaceutical precursors in a selective and environmentally friendly way via late-stage hydroxylation.

## 3. Materials and Methods

All chemicals were purchased from Sigma-Aldrich (Hamburg, Germany), Carl Roth (Karlsruhe, Germany), Merck (Darmstadt, Germany), or chemPUR (Karlsruhe, Germany), if not stated otherwise. Glucose dehydrogenase (GDH) from *Pseudomonas sp*. and catalase from bovine liver were obtained from Carl Roth. Salt-free oligonucleotides were obtained at HPSF purity from Eurofins MWG Operon (Ebersberg, Germany). DpnI and dNTPs were purchased from New England Biolabs (Frankfurt, Germany). *Pfu*S polymerase and *Taq* polymerase were produced in house. 

### 3.1. Strains, Plasmids, and Target Gene

The *Bacillus megaterium* strain ATCC 14581 codes for a self-sufficient fatty acid monooxygenase (CYP102A1, 118 kDa), more commonly known as P450 BM3 (WP_034650526.1. The epPCR and SSM libraries were cloned into expression vector pALXtreme-1a (derived pET-28a(+) vector) [57], and expression was achieved using *E. coli* BL21-Gold (DE3) lacI^Q1^ cells [57]. All oligonucleotides and primers used in this study are summarized in Table A1.

### 3.2. Error-Prone PCR 

The random mutagenesis library was constructed by the standard epPCR method [58]. In all PCRs, a thermal cycler (Mastercycler pro S; Eppendorf, Hamburg, Germany) and thin-wall PCR tubes (0.2 mL; Carl Roth GmbH, Karlsruhe, Germany) were used. DNA concentrations were quantified using a NanoDrop photometer (ND-1000, NanoDrop Technologies, Wilmington, DE, USA). The epPCR-P450_BM3_Heme library was generated by PCR (94 °C for 2 min, 1 cycle; 94 °C for 30 s/55 °C for 30 s/68 °C for 2 min, 25 cycles; 68 °C for 4 min, 1 cycle) using dNTP mix (0.2 mM), MnCl_2_ (0.05 mM and 0.075 mM), *Taq* polymerase (7.5 U, prepared in-house), plasmid DNA template (pALXtreme-1a-P450 BM3 WT, 5 ng), and primers (0.5 μM each, HPSF purity, Eurofins MWG Operon, Ebersberg, Germany) in a final volume of 50 μL. The vector backbone and reductase domain for the PLICing reaction [57] was generated by PCR (98 °C for 2 min, 1 cycle; 98 °C for 15 s/64 °C for 20 s/72 °C for 3 min, 25 cycles; 72 °C for 4 min, 1 cycle) using dNTP mix (0.2 mM), plasmid DNA template (pALXtreme-1a-P450 BM3 WT, 5 ng), *Pfu*S polymerase (3 U), and primers (0.5 μM each, HPSF purity, Eurofins MWG Operon, Ebersberg, Germany) in a final volume of 50 μL. The amplified epPCR products were digested (2 h, 37 °C) by *Dpn*I (20 U, New England Biolabs, Frankfurt, Germany) and then purified using the QIAquick^®^ PCR Purification Kit (QIAGEN, Steinheim, Germany), eluted with 35 μL ddH_2_O, and used for subcloning the insert DNA (Heme domain P450 BM3) into the vector (pALXtreme-1a with the reductase domain from P450 BM3) via PLICing reaction as published [57]. Subsequently, the constructs were transformed into chemically competent *E. coli* BL21 Gold (DE3) lacI^Q1^ cells. Plasmids were isolated using QIAprep^®^ Spin Miniprep Kit (QIAGEN, Steinheim, Germany), eluted with 35 μL ddH_2_O, and sent for sequencing to Eurofins MWG Operon (Ebersberg, Germany). SnapGene Software (GSL Biotech, Chicago, IL, USA) was used for sequence analysis.

### 3.3. Site Saturation Mutagenesis

The P450BM3WT-SSM library was generated by PCR. In all PCRs, a thermal cycler (Mastercycler pro S; Eppendorf, Hamburg, Germany) and thin-wall PCR tubes (Multi ultratubes; 0.2 mL; Carl Roth GmbH, Karlsruhe, Germany) were used. DNA concentrations were quantified using a NanoDrop photometer (ND-1000, NanoDrop Technologies, Wilmington, DE, USA). For each PCR (98 °C for 2 min, 1 cycle; 98 °C for 15 s/50–66 °C for 20 s/72 °C for 3 min, 25 cycles; 72 °C for 4 min, 1 cycle), we used dNTP mix (0.2 mM), plasmid DNA template (pALXtreme-1a-P450 BM3 WT, 5 ng), *Pfu*S polymerase (3 U) and NNK primers for positions 122, 255, 329 and 331 (0.5 μM each) HPSF purity (Eurofins MWG Operon, Ebersberg, Germany) in a final volume of 50 μL. The amplified PCR products were digested (2 h, 37 °C) by *Dpn*I (20 U, New England Biolabs, Frankfurt, Germany) and then purified using the QIAquick^®^ PCR Purification Kit (QIAGEN, Steinheim, Germany), eluted with 35 μL ddH_2_O. Subsequently, the constructs were transformed into chemically competent *E. coli* BL21 Gold (DE3) lacI^Q1^ cells. Plasmids were isolated using the QIAprep^®^ Spin Miniprep Kit (QIAGEN, Steinheim, Germany), eluted with 35 μL ddH_2_O, and sent for sequencing to Eurofins MWG Operon (Ebersberg, Germany). SnapGene Software (GSL Biotech, Chicago, USA) was used for sequence analysis.

### 3.4. Cultivation of P450 BM3 in 96-Deep-Well Plates

The cultivation of P450 BM3 in a 96-deep-well plate was done using an adapted protocol from Nazor et al. (2007). Single colonies of the P450 BM3 library were transferred into 96-well flat bottom MTPs (Greiner Bio-One GmbH, Frickenhausen, Germany) filled with LB medium (150 μL; 50 μg/mL kanamycin) using sterile toothpicks. Six wells of each MTP were inoculated with replicates of the negative control (empty vector) and the starting variant (P450 BM3 WT). Cultivation was performed in an MTP shaker (Multitron II; Infors GmbH, Einsbach, Germany) for 16 h (37 °C, 900 rpm and 70% humidity). The overnight cultures were used as pre-cultures for expression and stored at −80 °C after addition of 100 μL sterile glycerol (50% (*v*/*v*)). Library expression occurred in round bottom 2.2 mL 96-deep-well plates (Brand GmbH, Wertheim, Germany) in 600 μL of terrific broth (TB) medium (50 µg/mL kanamycin, 1 mM of IPTG, 100 mg/L thiamine hydrochloride, and 0.5 mM 5-aminolevulinic acid). Cells were incubated in an MTP shaker for 22–24 h (30 °C, 900 rpm, and 70% humidity). Expression cultures were harvested by centrifugation (15 min, 3220 *g*, 4 °C), the supernatant was discarded, and cell pellets were stored at −20 °C until further use.

### 3.5. Screening for Improved P450 BM3 Variants

Frozen cells were thawed on ice for 10 min and then resuspended in 300 μL KPi (50 mM, pH 7.5) supplemented with lysozyme (8 g/L) to disrupt them. An incubation for 1 h (37 °C, 900 rpm, and 70% humidity) followed, and lysed cells were centrifuged (20 min, 3220 *g* at 4 °C). Each MTP was analyzed in parallel using two different approaches, (a) using a variation of the 4-AAP assay for phenol-like product detection [43,50], and (b) using CE to investigate the formation of side products. We screened clones for increased hydroxylation. An NADPH depletion assay was performed as described by Glieder and Meinhold (2003) by measuring NADPH oxidation at λ 340 nm in a Tecan Sunrise MTP reader (Tecan Group AG, Männedorf, Switzerland). The reaction contained, per well: 50 μL cell lysate with expressed P450 BM3, 1.2 mM benzo-1,4-dioxane, 2% (*v*/*v*) EtOH, and KPi (50 mM, pH 7.5) in a total volume of 200 μL. MTPs were incubated for 5 min before supplementation with 50 μL NADPH (1 mM). Oxidation of NADPH was measured at λ 340 nm in a Tecan Sunrise MTP reader (Tecan Group AG). After NADPH depletion, 25 μL of a quenching solution was added (4 M urea in 0.1 M NaOH), then 20 μL of 4-aminoantipyrine (4-AAP) (5 mg/mL), and afterwards, 20 μL potassium peroxodisulfate (5 mg/mL) for phenolic-like product detection. Plates were incubated for 30 min at 500 rpm (room temperature). Absorbance was measured at λ 509 nm with a Tecan Sunrise MTP reader (Tecan Group AG, Männedorf, Switzerland). The standard deviation of the 4-AAP assay was determined using 92 replicates of P450 BM3 WT, and for the calculation of the true standard deviation, absorption values obtained for cell lysates without P450 BM3 (negative control, background) were subtracted. Additionally, and in parallel (for the rescreening only) using the same cell lysates from above, CE was used to investigate the formation of side products not detected by the 4-AAP assay. Briefly, each reaction contained, per well: 50 μL cell lysate with expressed P450 BM3, 1.2 mM benzo-1,4-dioxane, 2% (*v*/*v*) EtOH, 3 U/mL GHD, 1200 U/mL catalase, and 60 mM glucose in KPi (50 mM, pH 7.5) in a total volume of 200 μL. MTPs were incubated for 5 min before supplementation with 50 μL NADPH (1 mM) and left to react for 4 h (500 rpm, RT). Afterwards, 50 μL of a quenching solution (30 mM SDS, 15 mM NaPi, 6 mM benzyl alcohol in 4 M urea) were added and the plate centrifuged for 15 min, 3220 *g* at RT). Afterwards, 100 μL of the supernatant was transferred to a 96-well PCR plate (VWR, Atlanta, GA, USA) and sealed with a transparent film to avoid evaporation. Electrophoretic measurements were performed on 96 uncoated fused-silica capillaries (Advanced Analytical cePRO9600, Ames, IA, USA) equipped with a UV diode array detector set for 214 nm. Data acquisition was performed with pKa Analyzer v.1.2 (Advanced Analytical, USA). Prior to their first use, the capillaries were conditioned with 1 M NaOH and deionized water for 40 min, and before measurement, were conditioned with running buffer (30 mM SDS/15 mM NaPi) for 30 min. The capillary was flushed for 5 min with running buffer between runs. A pre-run at −11 kV for 1 min was followed by hydrodynamic sample injection (−0.70 psi for 45 s). Separation was performed applying a voltage of −11 kV for 40 min. The standard deviation of the electrophoretic measurements was determined using 92 replicates of active P450 BM3 WT.

### 3.6. Expression and Purification of P450 BM3 Variants

Shake flasks expression and purification of the P450 BM3 monooxygenase variants was performed adapting the original protocol described by Nazor et al. (2007) [59]. Briefly, for the purification, frozen cell pellets from a 250 mL culture were resuspended in 15 mL Tris/HCl buffer (100 mM, pH 7.5). Cells were homogenized by sonication for 5 min (with 30 s intervals, 50% amplitude, Vibra-Cell VCX-130; Sonics, Newtown, CT, USA). After centrifugation (30 min, 16,000 *g* at 4 °C), the supernatant was filtered with a 0.22 μm filter membrane. Purification of the P450 BM3 variants was performed by anion exchange chromatography with a Toyopearl DEAE 650S matrix (Tosoh Bioscience, Griesheim, Germany) and an ÄKTA prime chromatography system (GE Healthcare, Solingen, Germany) using a variation of the established protocol [60]. The purified P450 BM3 enzyme was concentrated with an Amicon centrifugation tube (50 kDa cut-off; Merck Millipore, Darmstadt, Germany) and desalted using a PD-10 desalting column (GE Healthcare) equilibrated with KPi (50 mM, pH 7.5). For long-term storage, enzyme samples were shock-frozen in liquid nitrogen and lyophilized (Alpha 1–2 LD plus freeze-dryer Christ, Osterode am Harz, Germany). For long-term conversions, cell-free lysates were used by resuspending the frozen cell pellets in KPi (50 mM, pH 7.5) (10% of culture volume) and lysed by sonication for 5 min (with 30 s interval, 50% amplitude, Vibra-Cell VCX-130). Cell debris was removed by centrifugation (30 min, 16,000 *g* at 4 °C).

### 3.7. Substrate Conversion and Kinetic Characterization of P450 BM3 Variants 

P450 BM3 concentrations were determined by CO-binding assay following the protocol by Omura and Sato (1964) [61]. Regioselectivity, product yields, and total turnover number (TTN) were determined, whenever standards for the products were available, in the presence of glucose dehydrogenase (GDH) for efficient regeneration of the NADPH cofactor. The TTN was determined with cell-free lysate and calculated based on 2,3 dihydrobenzo 1,4-dioxin-5-ol formation after 1 h of conversion. Conversions of 1 mL volume contained 1 μM P450 BM3 variant, 3 U/mL GDH, 60 mM glucose, 1200 U/mL catalase, 1.2 mM substrate, 2% (*v*/*v*) EtOH, 400 μM NADPH, and KPi (50 mM, pH 7.5). Kinetic characterizations were performed with purified P450 BM3. The reactions contained 1.2 mM benzo-1,4-dioxane and 2% (*v*/*v*) EtOH in a final volume of 1 mL KPi (50 mM, pH 7.5). After 5 min of incubation, NADPH was supplemented, and the oxidation of the cofactor was measured at λ 340 nm in a spectrophotometer (Varian Cary 50 UV). NADPH oxidation rates and coupling efficiencies were determined using 1 mM NADPH and 0.125–1 μM P450 BM3 (1 μM WT, 0.125 μM R255L, 0.125 μM R255G). The conversions were stopped with 100 μL 37% (*v*/*v*) HCl after respective reaction times (20 min) or after full depletion of NADPH. Products were extracted with 500 μL methyl *tert*-butyl ether (MTBE) containing 2 mM cyclododecanol as internal standard. Organic phases were dried over anhydrous MgSO_4_ and analyzed by GC-FID (gas chromatography with flame-ionization detector) (Shimadzu GmbH, Duisburg, Germany). Calibration curves were prepared with commercially available analytical standards. Products resulting from P450 BM3 conversions were separated using the following program: 100 °C for 1 min, heating 10 °C/min up to 200 °C, heating 20 °C/min up to 250 and holding for 10 min (Optima-17MS column, Macherey-Nagel). All reactions were performed in triplicate.

### 3.8. Molecular Modeling

#### 3.8.1. Molecular Docking

The starting coordinates of the P450 BM3 WT were taken from the crystal structure of cytochrome P450 BM3 with the heme domain (PDB ID: 1BU7 [54]). The models of the P450 BM3 variants R255G and R255L were constructed using the swap function in YASARA Structure Version 17.4.17 [62] and optimized using the SCWRL [63] rotamer library search for the designated substitutions. The protein residues were treated using the AMBER ff99 [64] and the substrate (benzo-1,4-dioxane) was treated employing the general amber force field (GAFF) [65,66] with AM1-BCC partial charges [67] with particle mesh Ewald [68] for long-range electrostatic interactions and a direct force cutoff of 10.5 Å. The crystal water molecules present in the crystal structure were deleted, except the one that is coordinated to the Fe^2+^ ion of the heme domain. The constructed models were minimized using a water box, first with steepest descent and then simulated annealing (timestep of 2 fs, atom velocities scaled down by 0.9 every 10 steps) starting from 98 K, 198 K, and 298 K with a time averaged Berendsen thermostat until convergence was reached. The minimized models were further used for molecular docking studies of the substrate benzo-1,4-dioxane. A grid box of 12 Å around the active site was applied by centering the heme iron of P450 BM3. Molecular docking calculations were performed using the Autodock4.2 plug-in within YASARA, with a fixed protein backbone. 100 docking runs were carried out, and the docking solutions were clustered applying an RMSD cutoff of 0.5 Å and using the default settings provided within the YASARA dock_run macro file. Molecular docking results were analyzed by considering the distance between the iron-bound water molecule and closest C atom (C5) of the benzo-1,4-dioxane substrate.

#### 3.8.2. Molecular Dynamics Simulations

Molecular dynamics simulations were carried out using the enzyme-substrate complex obtained from molecular docking of the substrate benzo-1,4-dioxane in the binding pocket of P450 BM3 WT and variants (R255G and R255L). The PROPKA 3.1 program [69] was used to determine the protonation states of titratable residues on the basis of pKa values and visual inspection. The amber ff14SB force-field parameters [70,71] for the protein and general amber force field (GAFF) [66] for heme were used. The required heme parameters were taken from the literature [72], and substrate benzo-1,4-dioxane was optimized with the B3LYP method [73] and 6-311G(d,p) [74] basis set using Gaussian09 [75]. Moreover, RESP charges were calculated using the Antechamber module in Amber14 [76]. The whole system was neutralized by adding 15 Na^+^ ions in WT and 16 Na^+^ ions in R255G and R255L variants. Hydrogen atoms were added using the tleap module of AmberTools14 [76]. The protein was solvated in an octahedral TIP3P water box centered at the center of mass to ensure a water layer of 12 Å around the protein. The systems contained ≈ 67,000 atoms in total, including ≈ 6623 TIP3P [77] water molecules. Initially, the solvent and the ions were minimized using whole-system minimization with 10,000 steps of steepest descent followed by 3000 steps of conjugate-gradient minimization. Afterwards, the system was heated slowly from 0 to 300 K for 50 ps. Constant pressure periodic boundary conditions using the particle mesh Ewald (PME) [68] method were employed during MD simulations. The electrostatic interactions were calculated using a cutoff of 10 Å. After the heating step, the systems were equilibrated for 1000 ps at 300 K. Three independent production runs, each for 50 ns, were carried out to have reasonable statistics. All classical molecular dynamics (MD) simulations were performed using the Amber14 program [76]. The obtained MD simulation trajectories were visualized and analyzed with Pymol [78], VMD [79], and AmberTools 14 [76].

## Figures and Tables

**Table 1:** Catalytic performance of benzo-1,4-dioxane conversion with purified P450 BM3 WT and variants R255G and R255L.

Variant	NADPH Oxidation Rate [min^−1^]	Coupling EFFICIENCY [%]	TTN
WT	8.3 ± 1.3	8.8 ± 0.1	40 ± 3
R255G	1719 ± 231	23.7 ± 0.5	798 ± 24
R255L	1168 ± 163	25.7 ± 1.0	860 ± 15

NADPH oxidation rate (min^−1^) was determined spectrophotometrically at λ 340 nm; coupling efficiency (%) = ratio between 2,3-dihydrobenzo-1,4-dioxin-5-ol formation [μM] and oxidized cofactor [μM]. Nicotinamide adenine dinucleotide phosphate (NADPH) oxidation rate and coupling efficiency were determined using purified P450 BM3. The reaction was supplemented with 1 mM NADPH, and the activity of P450 BM3 was measured as initial NADPH oxidation rates at λ 340 nm. The total turnover number (TTN) was determined with cell-free lysate and calculated based on 2,3-dihydrobenzo-1,4-dioxin-5-ol formation after 1 h. Products were quantified using GC-FID and commercial standards. All reactions were performed in triplicate.

**Figure A8:**
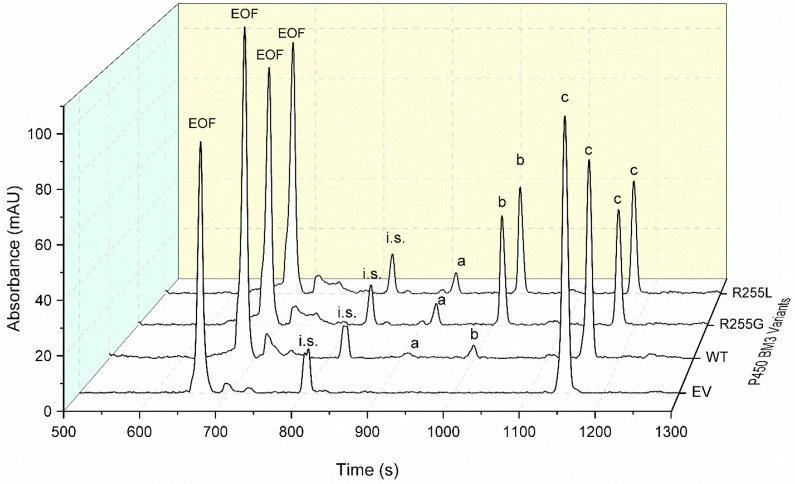
Product analysis via CE after conversion of benzo-1,4-dioxane with lysate from EV (negative control), P450 BM3 WT, and variants R255G and R255L. The data was obtained from 4 h conversion reactions employing GDH for efficient NADPH cofactor regeneration. EOF—electro-osmotic flow; i.s.—internal standard (benzyl alcohol); a—2,3-dihydrobenzo-1,4-dioxin-6-ol; b—2,3-dihydrobenzo-1,4-dioxin-5-ol; c—benzo-1,4-dioxane.

## Abbreviations

AAP	Aminoantipyrine
BM3	*Bacillus megaterium* 3
CE	Capillary electrophoresis
CO	Carbon monoxide
EV	Empty vector
FAD	Flavin adenine dinucleotide
FID	Flame ionization detector
FMN	Flavin mononucleotide
HPSF	High purity salt free
GC	Gas chromatography
GDH	Glucose dehydrogenase
IPTG	Isopropyl β-D-1-thiogalactopyranoside
KPi	Phosphate buffer
MD	Molecular dynamics
MS	Mass spectroscopy
MTBE	Methyl *tert*-butyl ether
MTP	Microtiter plate
NADPH	Nicotinamide adenine dinucleotide phosphate
NaPi	Sodium phosphate buffer
P450	Cytochrome P450 monooxygenase
PCR	Polymerase chain reaction
RESP	Restrained Electrostatic Potential
RMSD	Root mean square deviation
RMSF	Root mean square fluctuation
SSM	Site saturation mutagenesis
TTN	Total turnover number
WT	Wild type

## Appendix A

**Table A1:** List of oligonucleotides and primers used for cloning, sequencing, and mutagenesis during this work.

Name	Target	Sequence (5′–3′)
P450_BM3_FWD	P450 BM3 Heme	catgggcatGACAATTAAAGAAATGCCTCA-
P450_BM3_REV	P450 BM3 Heme	gcgtattatgaGCGTTTTCTGCCTTTTTGC
pALX_FWD	pALXtreme-1a with P450 BM3 reductase	ctcataatacGCCGCTGCTTGTGCTATACG
pALX_REV	pALXtreme-1a with P450 BM3 reductase	gcgtattatgAGCGTTTTCTGCCTTTTTGC
122.FWD	Position 122	ATGATGGTCGATNNKGCCGTGCAGCTT
122.REV	Position 122	AAGCTGCACGGCMNNATCGACCATCAT
255.FWD	Position 255	GACGAGAACATTNNKTATCAAATTATT
255.REV	Position 255	AATAATTTGATAMNNAATGTTCTCGTC
329.FWD	Position 329	TGGCCAACTGCTNNKGCGTTTTCCCTA
329.REV	Position 329	TAGGGAAAACGCMNNAGCAGTTGGCCA
331.FWD	Position 331	ACTGCTCCTGCGNNKTCCCTATATGCA
331.REV	Position 331	TGCATATAGGGAMNNCGCAGGAGCAGT

Capital letters: standard nucleotides connected through phosphorodiester bonds; lower case letters: nucleotides connected through a phosphorothioatediester bond; N: A, C, G, T; K: G, T.
